# Characterization of Lactate Sensors Based on Lactate Oxidase and Palladium Benzoporphyrin Immobilized in Hydrogels

**DOI:** 10.3390/bios5030398

**Published:** 2015-07-07

**Authors:** Liam P. Andrus, Rachel Unruh, Natalie A. Wisniewski, Michael J. McShane

**Affiliations:** 1 Department of Biomedical Engineering, 5045 Emerging Technologies Building, 3120 TAMU, Texas A&M University, College Station, TX 77843, USA; E-Mails: lpa6@tamu.edu (L.P.A.); rachel_unruh@tamu.edu (R.U.); 2 PROFUSA, Inc., 345 Allerton Avenue, South San Francisco, CA 94080, USA; E-Mail: natalie.wisniewski@profusa.com; 3 Department of Materials Science and Engineering, 3003 TAMU, Texas A&M University, College Station, TX 77843, USA

**Keywords:** luminescence, lactate, enzyme, biosensor, hydrogel

## Abstract

An optical biosensor for lactate detection is described. By encapsulating enzyme-phosphor sensing molecules within permeable hydrogel materials, lactate-sensitive emission lifetimes were achieved. The relative amount of monomer was varied to compare three homo- and co-polymer materials: poly(2-hydroxyethyl methacrylate) (pHEMA) and two copolymers of pHEMA and poly(acrylamide) (pAam). Diffusion analysis demonstrated the ability to control lactate transport by varying the hydrogel composition, while having a minimal effect on oxygen diffusion. Sensors displayed the desired dose-variable response to lactate challenges, highlighting the tunable, diffusion-controlled nature of the sensing platform. Short-term repeated exposure tests revealed enhanced stability for sensors comprising hydrogels with acrylamide additives; after an initial “break-in” period, signal retention was 100% for 15 repeated cycles. Finally, because this study describes the modification of a previously developed glucose sensor for lactate analysis, it demonstrates the potential for mix-and-match enzyme-phosphor-hydrogel sensing for use in future multi-analyte sensors.

## 1. Introduction

Sensors capable of rapid and accurate analyte detection are essential to the future of personalized medicine [1,2]. Further, the ability to track and record *in vivo* substrate concentrations in real time will enable more timely, precise diagnosis and improved management of chronic conditions [3,4]. Currently, a blood sample is collected and analyzed *ex vivo* using an electrochemical enzyme assay. Even with the advent of portable, handheld, and point-of-care devices, patients endure painful, repeated fingersticks and the limitations of discrete time-point analysis do not provide caregivers or patients the ability to monitor systemic fluctuations over time. A prime example of this is diabetes, where knowledge of blood glucose dynamics helps inform decisions about treatment and lifestyle; yet, there are other situations where tracking biochemistry may also lead to improved outcomes.

For example, l-Lactate is an analyte of great interest due to its role in sports medicine [5], clinical chemistry [6], the food processing industry [7], and overall normal metabolic function. While lactate is a normal byproduct of cellular metabolism, intracellular concentrations of lactate increase more dramatically during anaerobic respiration; likewise, interstitial lactate levels increase as it is excreted by cells, and it can accumulate in muscles and other tissues to cause soreness, pain, and impaired function [8,9]. Therefore, lactate can be used to assess a variety of acute deoxygenation events including hypovolemia (shock) [10], heart disease [11], and renal failure [12]. High lactate levels are also commonplace in traumatic injury, where a patient has undergone significant blood loss. In fact, monitoring of blood lactate levels is shown to improve identification of patients requiring resuscitative care (93%) when compared to standard blood pressure monitoring (67%) [13]. Thus, continuous monitoring of lactate is attractive for dynamic health assessment of active-duty military and other high-risk personnel. Additionally, lactate levels can be used to approximate “oxygen debt” in endurance athletes [14], allowing for optimal training in their chosen endeavor. These facts highlight the use of continuous lactate monitoring for systemic health analysis in military personnel and other critical care situations [13,15].

Although there have been recent advances in continuous analyte monitoring systems, there still remains a dearth of long term, minimally invasive options. To date, there have been a number of attempts to develop miniaturized platforms for continuous monitoring of various biochemical metabolites [16,17,18,19,20]. Many of these devices rely on an electrochemical-sensing assay for metabolite detection. For example, a successful system uses an enzyme to produce hydrogen peroxide (H_2_O_2_) at the surface of an implanted electrode and the resulting change in electric potential can be correlated to interstitial lactate concentrations [21].

While electrochemical biosensors represent a major advancement in continuous interstitial analyte monitoring, they still experience a multitude of technical challenges to effective long-term use [22,23,24,25]. The entry wound caused by implantation elicits an inflammatory response from surrounding tissue. Macrophage recruitment eventually gives way to fibrotic encapsulation, restricting substrate diffusion at the tissue/electrode surface. These changes in sensitivity result in a reduction in the apparent sensitivity, requiring multiple recalibrations and eventually leads to shortened sensor lifetime, even though the sensors themselves retain function. In addition, the implantation site provides a potential bacterial pathway, leading to an increased risk of infection.

Optical sensing techniques display many advantages over electrochemical-based methods. Optical systems rely on photon transfer and detection, eliminating need of a transcutaneous connection. Previous work has shown the feasibility of using dual wavelength polarimetry [26], optical coherence tomography [27], and Raman spectroscopy [28] as techniques for metabolite detection and protein-protein interactions. Those studies, and many failed commercialization efforts, have also revealed the weakness of purely noninvasive approaches; they are not sensitive or selective enough to function effectively in real world situations.

As with optical glucose sensors, reports on optical lactate detection primarily use fluorescent indicator molecules [29,30] or spectroscopic [31] techniques. Molecular recognition agents provide a degree of selectivity for the target analyte, and luminescence detection dramatically improves sensitivity; the combination of these two properties results in “transducers” to convert chemical concentration into a fluorescent or phosphorescent signal [32]. The McShane group developed enzymatic glucose sensors based on microparticles and microcapsules with specialized diffusion-limiting coatings; Kazakova *et al.* developed lactate sensitive micro-capsules with a similar approach, using a pH-sensitive fluorophore for detection [29]. In a similar approach, Hu *et al.* developed highly fluorescent cupric oxide nano-particles functionalized with terephthalic acid, a substrate in peroxidase catalysis. This allows for enzyme mimetic characteristics, albeit with more stable activity than seen in natural peroxidase. By coupling this H_2_O_2_ sensing system with a lactate consuming enzyme, a highly fluorescent lactate nano-sensor was created [30].

The aforementioned optical sensing modalities rely on changes in photon intensity and are thus susceptible to tissue scattering effects as well as variations in temperature, pH and other physiological variables. Both lactate sensors suffered from low effective ranges and their long-term stability was not evaluated, and reversibility is not possible in the peroxide-coupled system. Further, neither nano-particles nor micro-capsules demonstrated potential for use as an implantable device for continuous monitoring *in vivo*.

Recently, highly sensitive phosphors have been developed and applied for biomolecular assay applications [22]. Some of these molecules are sensitive to oxygen quenching. In ambient conditions, emission intensities remain low due to de-excitation via energy transfer to O_2_ by collisional quenching. As O_2_ is reduced, phosphorescent intensities and emission lifetimes increase significantly. This quenching behavior is described by the Stern-Volmer relationship.

(1) $$\frac{{\text{τ}}_{0}}{\text{τ}}=\frac{I_{0}}{I}=1+K_{sv}\left[{\text{O}}_{2}\right]$$

where τ_0_ and *I*_0_ are phosphorescent lifetime and intensity of phosphor in absence of O_2_, τ and *I* are lifetime and intensity, *K_sv_* is the phosphor specific Stern-Volmer constant, and [O_2_] is oxygen concentration. Benzoporphyrin-based oxygen indicators absorb and emit at wavelengths within the commonly termed “optical window”, allowing for relatively efficient photon transmission through skin [33,34,35,36]. Additionally, phosphors are characterized by relatively long emission lifetimes (single to hundreds of microseconds), compared to tissue fluorophores (few nanoseconds); this property can be utilized to effectively discriminate sensor emission from background and natural fluorescence using straightforward signal processing techniques. Thus, emission lifetime can be used *in lieu* of intensity as a means to sensitively interrogate implants with minimal interference.

Oxygen-sensitive phosphors can be exploited for alternative substrate analysis by co-localization with an oxidoreductase enzyme. One example, lactate oxidase (LOx), uses both O_2_ and *lactate* as its co-substrates. Kinetics of LOx are described in the following reaction equations.

(2) $$\begin{array}{c} E_{ox}+L\overset{k_{1}}{\Rightarrow }E_{red}X_{1}\overset{k_{2}}{\Rightarrow }E_{red}+P\\ E_{red}+{\text{O}}_{2}\overset{k_{3}}{\Rightarrow }E_{ox}^{*}X_{2}\overset{k_{4}}{\Rightarrow }E_{ox}+{\text{H}}_{2}{\text{O}}_{2} \end{array}$$

where *E_ox_* and *E_red_* are the oxidized and reduced form of LOx, *L* is lactate, *P* is pyruvate, *E_red_X*_1_ is reduced enzyme-substrate complex, E*_ox_ X_2_ is oxidized enzyme-substrate complex, and *k*_1_, *k*_2_, *k*_3_, *k*_4_ are reaction rate constants (only forward constants are shown here) [37]. Incoming lactate increases catalysis and consequently oxygen consumption. Less collisional quenching by molecular O_2_ leads to longer emission lifetimes of the co-localized phosphor. Thus, approximation of local lactate levels is possible by monitoring phosphor quenching kinetics in an enzyme-controlled O_2_ micro-environment.

One key to using this concept to create implantable sensor devices is the encapsulation of the enzyme and phosphor sensing components into an appropriate biomaterial matrix, such as a hydrogel [38]. Poly(2-hydroxyethyl methacrylate) (pHEMA) is a synthetic polymer used in numerous biomedical devices, most notably soft contact lenses [39], drug delivery systems [40,41], and tissue engineering constructs [42]. pHEMA is attractive for use in implantable devices due to its low cytotoxicity and biofouling properties [43]. pHEMA’s ability to swell in water without losing mechanical integrity makes it an ideal material for *in vivo* applications [44,45,46,47]. Similarly, poly(acrylamide) (pAam) has many uses, initially purposed as a separation medium for gel electrophoresis applications [48,49]. Like pHEMA, pAam’s hydrophilic nature and low biofouling properties make it suitable for *in vivo* applications [49]. Additionally, both pHEMA and pAam have been investigated for immobilization of enzymes and other indicator molecules in biosensing applications [50,51,52]. Cross-linking enzymes within hydrogels has shown to enhance catalytic stability [53,54,55]. Thus, homogenous distribution of sensing chemistry within a biocompatible gel is attractive for enzyme based sensing applications.

This paper reports on *in vitro* characterization of a hydrogel based phosphorescent biosensor for lactate determination, where the system is designed to meet the needs described above. The studies herein focus on determining the effects of co-localizing LOx and an oxygen-sensitive phosphor within a hydrogel matrix, particularly on the oxygen and lactate diffusion, dose-response of the entire system to lactate (oxygen depletion), and finally the stability of the sensors to repeated cycling. This builds upon our previous efforts with glucose sensing, illustrating the general applicability of the enzyme-benzoporphyrin-hydrogel platform and how it may be used to take us toward the ultimate goal of multianalyte detection.

## 2. Experimental Section

### 2.1. Materials

Catalase, ethylene glycol, 2,2-dimethoxy-2-phenyl-acetophenone (DMAP), 1-Ethyl-3-[3-dimethylaminopropyl]carbodiimide hydrochloride (EDC), and sodium lactate were purchased from Sigma-Aldrich^®^ (St. Louis, MO, USA). Dimethyl sulfoxide (DMSO) was purchased from VWR^®^ (Radnor, PA, USA). Sodium chloride, potassium phosphate (dibasic), and potassium chloride were purchased from VWR (Radnor, PA, USA). Sodium phosphate (monobasic) was purchased from ACROS Organics (ThermoFisher Scientific Inc.™, Waltham, MA, USA). 2-hydroxyethyl methacrylate (HEMA) and tetra(ethylene glycol) methacrylate (TEGDMA) were purchased from Polysciences Inc. (Warrington, PA, USA). Lactate oxidase from *Aerococcus viridians* (LOx), acrylamide (Aam), and sulfo-*N*-hydroxysuccinimide (sulfo-NHS) were purchased from A.G. Scientific™ (San Diego, CA, USA), AMRESCO^®^ (Solon, OH, USA), and G Biosciences™ (St. Louis, MO, USA) respectively. Palladium (II) tetramethacrylated benzoporphyrin (BMAP) was donated by PROFUSA Inc., (San Francisco, CA, USA). All chemicals are reagent grade and used without further purification.

### 2.2. Sensor Preparation 

To synthesize gels, 2.5 mg DMAP was weighed into a micro centrifuge tube. Aam was dissolved in DMSO in a 67.2 *v*/*v* % concentration. Two hundred and fifty microliters of monomer precursor (containing the proper *v*/*v* % ratio of HEMA to Aam solution plus 5 µL TEGDMA) was added to DMAP and vortexed. Next, 90 µL ethylene glycol was added as a co-solvent and vortexed again. Fifty microliters of 10 mM BMAP solution in DMSO was added along with 125 µL LOx/Catalase solution (pH = 7.4) in a 10:1 molar ratio. Both dye and enzyme were repeatedly pipetted to ensure proper mixing. Sensors were made with three different co-polymer materials: 75:25 pHEMA:pAam, 90:10 pHEMA:pAam and pure pHEMA. Resulting solutions were pipetted into a premade mold (consisting of a 0.03ʹʹ spacer sandwiched between two clean microscope slides) and exposed to UV light to induce polymerization. Gels were removed from molds and placed into PBS solution containing 15 mg sulfo-NHS and 6.6 mg EDC. To ensure maximum enzyme cross-linking within gels, sensors were allowed to react overnight. Afterwards, gels were rinsed with DI water, placed in fresh PBS, and stored at 4 °C in foil to prevent photobleaching. Sensors were cut into 5 mm strips rinsed prior to testing.

### 2.3. Benchtop Testing System

**Figure 1:**
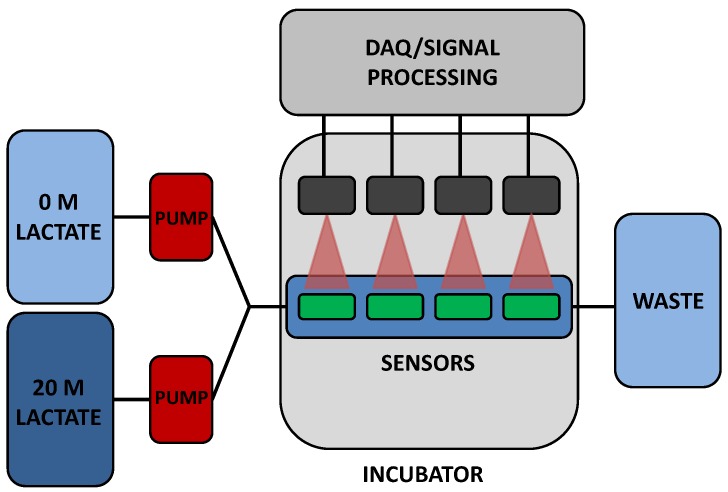
Flow through system schematic.

To validate *in vitro* sensor response, a custom flow-through system was used. Two positive displacement VICI^®^ M6 liquid pumps (Valco Instruments Co., Inc., Houston, TX, USA) are connected to reservoirs containing either a highly concentrated (20 mM lactate) solution or a PBS solution (0 mM lactate). A LabVIEW™ program controls flow rates from both pumps independently. Flow from each reservoir is mixed prior to reaching a specially designed cell in which four sensors are immobilized. In a typical experiment, three lactate sensors and a pHEMA-BMAP oxygen sensor were placed in the flow cell for simultaneous monitoring. Lactate concentration was modulated throughout each experiment and resulting lifetime data was collected and stored for later analysis. It was deemed impractical to test sensors in blood or serum at this time, due to contamination of pumps in addition to the volume of solutions needed. While the conditions used for testing do not completely represent the *in vivo* expected situation, they should be considered adequate for the initial characterization of this new type of sensor system. A schematic of the flow through system is shown in Figure 1.

To interrogate immobilized BMAP, a custom optical instrument was used. The excitation component of the setup is a Phillips^®^ Lumileds Luxeon Rebel red excitation source with a peak at λ_typ_ = 627 nm and a bandwidth of ≈20 nm. The diode has a 125° viewing angle, common among most LEDs, and is operated at 200 mA. Upon phosphor excitation, emitted photons are collected with a ball lens situated at 8 mm distance from the LED port; this light is collimated and passed through a Semrock™ FF02-809/81 filter prior to detection with a Si PMT (SensL). The entire system is contained within a miniaturized 1.5ʹʹ diameter plastic casing; each such “reader head” interrogates a single sensor of interest. A LabVIEW™ program controls LED intensity, pulse on/off parameters, data acquisition, and emission lifetime calculation from the measured decays as described below.

### 2.4. Data Analysis and Sensor Response

Emission lifetime of immobilized phosphor was measured as decay in voltage signal across a PMT detector. Immediately following a 500 μs LED pulse, peak intensity was measured. Sampling of voltage was delayed for 7 μs after pulse to reduce interference from the LED excitation pulse. Background voltage was calculated as the average intensity of the last 20% of the decay duration (2500 μs). Acquisitions were repeated quickly, generally taking 1.3 seconds per cycle. Emission lifetime decay curves were found by averaging 128–256 consecutive sets of intensity decays. Decay signal is defined from *t* = 507 μs to *t* = 2500 μs. Data were fit to an exponential defined in Equation (3). From this a value for phosphorescent lifetime (τ) may be obtained.

(3) $${\left[Q\right]}_{t}={\left[Q\right]}_{0}{\text{e}}^{-t/\text{τ}}$$

where [*Q*]*_t_* and [*Q*]_0_ are concentrations of excited state molecules at time *t* and time 0, respectively, and τ is the phosphorescent lifetime. Excited photons are lost due to various radiative and non-radiative processes. This makes the total phosphorescent lifetime a sum of each individual contribution.

A number of commonly used performance metrics were used to assess sensor response. After exposing sensors to an initial lactate challenge, adjustments were made to modulate sensors within a more appropriate range. Specifically, the lactate concentration steps used in the challenges were adjusted to provide for calibration curves with many points where the responses were most sensitive. Analytical range, sensitivity, and response times were calculated from calibration curves of *n* = 3 sensors. Analytical range is the span over which sensors accurately detect changes in lactate concentration, defined as the interval between upper and lower limits of detection (LOD_high_/LOD_low_). LOD_low_ is defined as emission lifetime seen at 0 mg/dL lactate plus (+) 3 standard deviations. Similarly, LOD_high_ is defined as emission lifetime at enzyme saturation minus (−) 3 standard deviations. Sensitivity is defined as the linear slope of each calibration curve, while response time refers to lag between lactate input and steady sensor response. Each point on a calibration curve represents a steady lifetime response over 30 min for a given lactate concentration. While steady response was observed within the first twenty minutes of a step increase in lactate, we allowed sensors to be exposed to each concentration for 1 h in order to collect more data points, thus providing for more robust data analysis. Sensors were subjected to a flow rate of 4 mL/min. “Flight plans” are progressively higher lactate challenges run consecutively on sensors before a return to baseline. Unless otherwise noted, all lactate challenges were performed at 37 °C.

### 2.5. Diffusion Analysis

To assess lactate transport, a horizontal diffusion cell system was used, following procedures to those detailed in previous work [52]. Briefly, hydrogels were prepared as described in Section 2.2, except that polymerization was performed using a 0.01ʹʹ glass mold; the thinner samples allowed for more rapid experimentation. Sensors were preconditioned in a 60 °C water bath for 10 days prior to diffusion study in order to completely deactivate immobilized LOx. LOx deactivation was confirmed by monitoring phosphorescent signal during a single lactate challenge. Deactivation of LOx mitigates lactate consumption concerns and allowed for a more reliable analysis of diffusion kinetics.

Each 7-mm diameter gel cutouts was sandwiched between two 7 mL reservoirs. Feeder reservoirs contain 1 M lactate solution, while permeate reservoirs contain pure PBS solution. To determine the rate of concentration change, *dc*/*dt*, permeate chambers were sampled for lactate over several hours using a YSI™ 2700 Select Biochemistry Analyzer (YSI Inc™, Yellow Springs, OH, USA); *dc*/*dt* was estimated as the slope of a linear fit to the concentration data. To calculate a relative diffusion coefficient, *D_L_*, Fick’s 2nd law of diffusion was used, with the assumption that *c_lac_*(0) *=* at *t*(0) in the permeate reservoir and homogenous mixing in each,

(4) $$D_{L}=\frac{dc}{dt}\left(\frac{bV}{\emptyset }\right)$$

where *D_L_* is the diffusion coefficient of lactate in cm^2^/s, *dc*/*dt* is change in permeate reservoir concentration over time, b is thickness of gel, *V* is the ratio of volume of reservoir to area of gel exposed to solution, and ϕ is the partition coefficient (assumed to be 1). Every material type was tested in triplicate at 25 °C and *D_L_* is reported as an average.

O_2_ transport was investigated by analyzing quenching kinetics of immobilized BMAP. Using Equation (1), material specific *K_sv_* values were calculated. Six hundred milliliters of PBS solution is connected to a flow cell containing sensors. Dissolved O_2_ concentration in PBS solution is controlled with a nitrogen bubbling system. A 1179A, MKS mass flow controller and a pressure gauge controller model PR 4000F, MKS, (MKS Instruments, Andover, MA, USA) are used to control flow of nitrogen gas and compressed air to the bubbler probe. O_2_ was decreased in a stepwise manner at concentrations of 21%, 10.5%, 5.25%, and 2.1%. Afterwards, 0% O_2_ was achieved by loading flow cell with 4 M glucose and 30 μM GOx solutions. The flow cell was then sealed and the mixture was allowed to react overnight. The resulting reaction consumes all local O_2_, allowing for detection of τ_0_. Stern-Volmer constants, *K_sv_*, were calculated from a linear fit to the calculated τ_0_/τ. Each sensor type was tested in triplicate at 37 °C.

### 2.6. Acute Sensor Degradation

To test for acute enzyme degradation, sensors were subjected to 20 consecutive lactate challenges. Flight plans were normalized in order to maintain a similar rate of enzymatic consumption within each material, meaning concentrations used were determined for each sensor type based on the upper limit of detection. Interrogations are step increases in lactate concentration before a return to baseline as seen in Section 2.4; however flight plans modulate between 0 mg/dL and the predetermined LOD_high_ of each sensor iteration. “Flight plans” were executed in series, with a return to 0 mg/dL lactate in between each. Afterwards, signal loss was calculated as percent change in emission lifetime from the initial (cycle 1) flight plan. Percent change was calculated at LOD_high_ as well as at intermediate concentrations.

## 3. Results and Discussion

### 3.1. Diffusion Analysis

**Figure 2:**
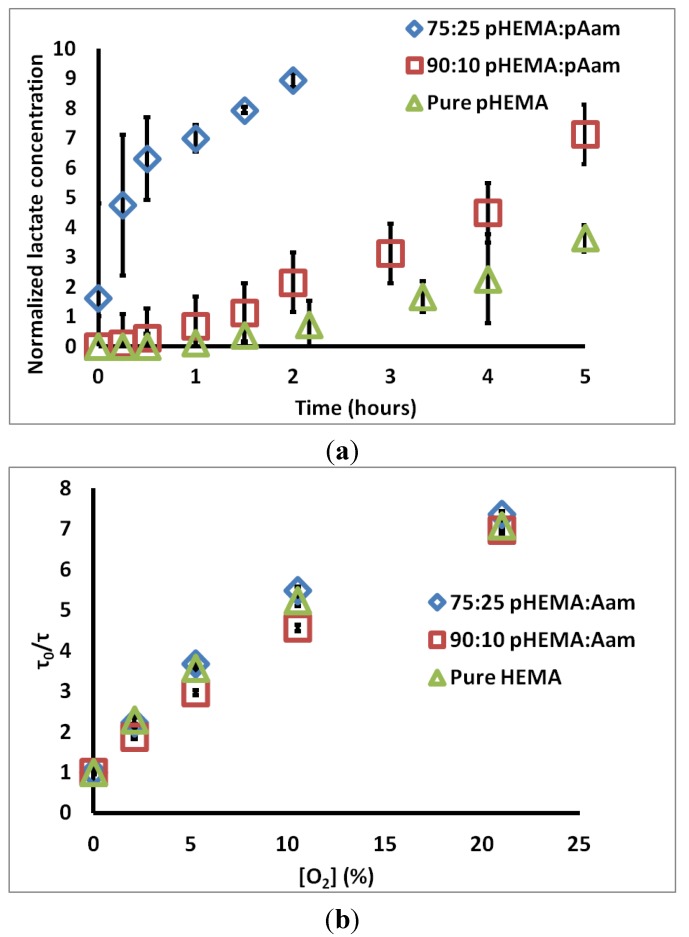
(**a**) Change of lactate concentration in permeate chamber over time for three sensor types; (**b**) Stern-Volmer plots for the same sensor types. Each set is an average of three compositionally identical sensors; errors bars denote 95% confidence intervals.

Figure 2a contains the measured permeate chamber lactate concentration for the three sensor types. Qualitatively, the increase in lactate transport through gels containing acrylamide is obvious. The calculated relative *D_L_* values for 75:25 pHEMA:pAam, 90:10 pHEMA:pAam, and pure pHEMA are 5.68 ± 0.323 × 10^−7^, 3.98 ± 0.97 × 10^−7^, and 3.13 ± 1.62 × 10^−7^ cm^2^/s, respectively. 90:10 pHEMA:pAam sensors display a small increase in lactate transport when compared to pure pHEMA, suggesting more pAam is needed in order to substantially effect swelling properties. For 75:25 pHEMA:pAam sensors, a ≈2-fold increase in lactate diffusion is seen relative to pHEMA sensors.

Figure 2b contains τ_0_/τ *vs.* [O_2_] plots for each material tested. *K_sv_* values for 75:25 pHEMA:pAam, 90:10 pHEMA:pAam, and pure pHEMA are 0.29 ± 0.003 (% O_2_)^−1^, 0.28 ± 0.01 (% O_2_)^−1^, and 0.28 ± 0.002 (% O_2_)^−1^, respectively. These *K_sv_* values match well with previous studies on similar materials [35]. Only the pure pHEMA and 75:25 formulations were statistically different at the 95% confidence level; this difference, while statistically significant, is only a matter of 3.6% increase in oxygen quenching. Thus, the effects on oxygen diffusion are minimal. This is not surprising, as O_2_ is a very small, hydrophobic molecule with low solubility in water. Thus, transport may depend less on material cross-link density and more on the O_2_ favorability of each polymer. It is most important to appreciate here that the changes in polymer formulation dramatically change lactate diffusion while minimally altering oxygen diffusion. Thus, this particular combination of polymers allows tuning of lactate diffusion almost completely independently from oxygen.

### 3.2. Sensor Response

**Figure 3:**
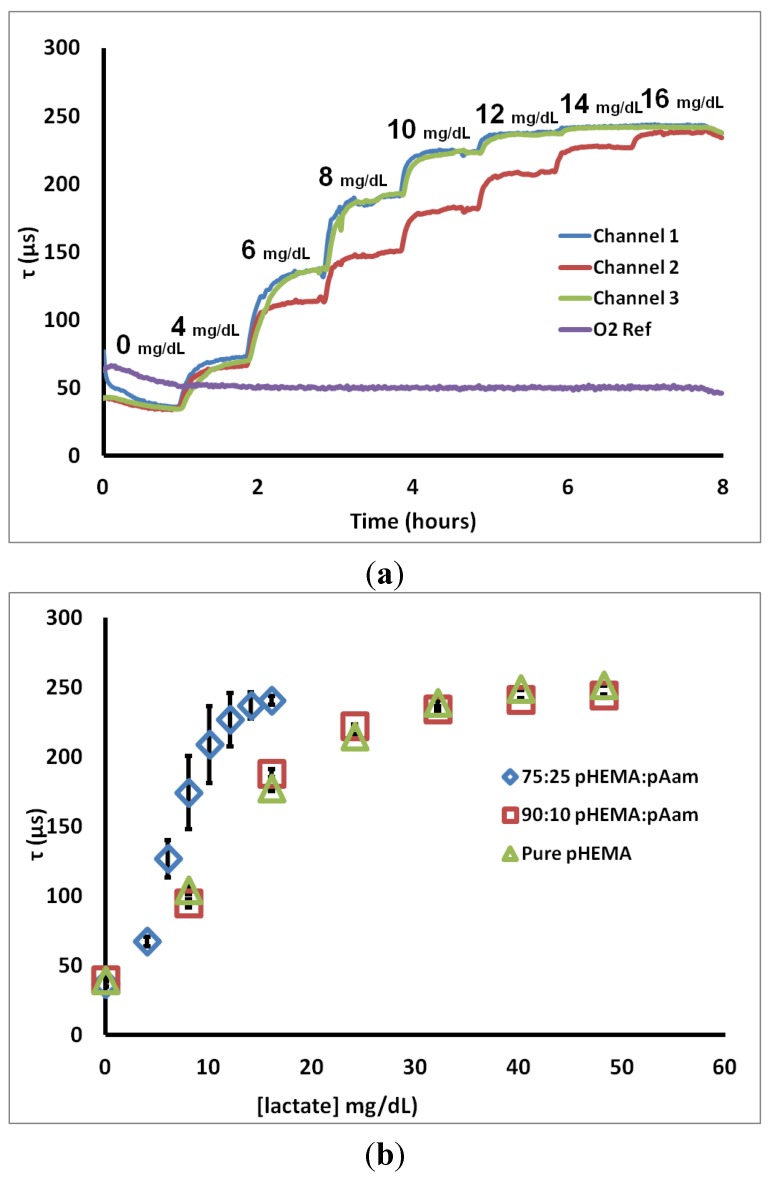
(**a**) 75:25 pHEMA:pAam lifetime response to lactate interrogation (**b**) calibration curves for three sensor types. Each calibration curve contains points representing the average phosphorescent lifetime; error bars denote the 95% confidence intervals for *n* = 3 sensors.

To determine characteristic sensor response, lactate challenges were administered. Figure 3a contains a representative real-time “flight plan” plot of the change in phosphorescent lifetime for three 75:25 pHEMA:pAam sensors to progressively higher lactate concentrations. The observed stepwise response is common to all formulations, regardless of composition. Most sensors matched well with others from the same batch, while a few cases (such as Channel 2 in Figure 3a) were significantly different in their response to intermediate lactate concentrations. All lactate sensors plateaued at a maximum lifetime between 225 and 250 µsec. A fourth trace represents the response of the oxygen sensor placed in the same channel, indicating the stable oxygen level observed in the steady state even during lactate challenges.

Figure 3b shows calibration curves representing the steady-state response to lactate for sensors based on the three different hydrogel types. Several points can be made from these data. First, all of the formulations yield the same lifetime at zero lactate, again reinforcing that the oxygen diffusion properties are essentially the same for each case. It is also immediately apparent that the incorporation of 25% acrylamide dramatically shifts the sensor behavior from the other two cases. First, the sensitivity to lower lactate levels was increased, while the lactate concentration at which lifetime nears the maximum (LOD_high_) was cut in approximately half. Furthermore, the amount of variability between sensors increased significantly, as indicated by the larger confidence intervals. This increased variability is primarily a result of batch heterogeneity, which was observed visually in preparing the hydrogels. The increased acrylamide content resulted in an obvious increase in gel phase separation/heterogeneity. Thus, sensors cut from the same initial hydrogel slab are more inconsistent in appearance. This apparent difference likely results in the performance difference, which we attribute to variability in localized enzyme concentration and diffusion properties in the acrylamide-containing gels. This heterogeneity is less pronounced for the 90:10 copolymers. It should be noted that a number of options exist to reduce the batch variability seen herein. Use of solvent mixtures that improve miscibility of the complex precursor, thereby reducing aggregation/phase separation/precipitation of the sensing chemistry during the mixing and polymerization process, is one option. Additionally, enzyme modification techniques (*i.e.*, PEGylation, modification of glycosylation sites with more hydrophilic or hydrophobic species) may be performed in order to allow for dissolution in alternative solvents without substantial activity loss. Lastly, precipitates may be filtered out prior to polymerization, if observed in the precursor mixtures. This latter option is an inefficient but viable solution.

Corresponding sensor figures of merit and diffusion metrics for the 75:25 pHEMA:pAam, 90:10 pHEMA:pAam, and pure pHEMA materials are reported in Table 1. These numbers quantitatively support the notion that increasing pAam precursor ratios are correlated to a decrease in range and a corresponding increase in sensitivity. This inverse relationship is explained by the properties of the two polymers. pAam is known to be significantly more hydrophilic than pHEMA due to polar amide groups present in pAam’s primary structure. In fact, pAam is able to take on ≈80% its weight in aqueous solution (compared to ≈37% for pHEMA) [36]. Higher concentrations of pAam lead to a more loosely cross-linked matrix, and therefore a more rapid diffusion profile, as is clearly seen from the measured relative lactate diffusion values. In contrast, however, previous reports cited a much larger increase of diffusion when comparing pAam to pHEMA; diffusion of small molecules was been shown to be several orders of magnitude higher in pAam when compared to pure pHEMA [52]. Thus, the copolymer system retains a strong influence of the pHEMA even with 25% pAam.

**Table 1:** Compiled sensor metrics, values are average of three sensors ±95% confidence intervals.

Monomers	75:25 pHEMA:pAam	90:10 pHEMA:pAam	Pure pHEMA
Sensitivity (μs × dL/mg)	19.0 ± 2.3	9.2 ± 1.5	8.5 ± 2.2
Range (mg/dL)	1.1–12.7	0.7–35.0	0.4–38.2
Response time (min)	19.0 ± 2.9	16.4 ± 1.7	15.2 ± 1.2
*D_L_* (cm^2^/s) × 10^−7^	5.7 ± 0.3	4.0 ± 0.9	3.1 ± 1.6
τ_0_ (μs)	251.8 ± 5.1	259.9 ± 8.9	290.0 ± 7.3
*K_sv_* (%^−1^ O_2_) × 10^−2^	29.4 ± 0.3	28.0 ± 1.2	27.7 ± 0.2
Signal retention @ LOD_high_ (%)			
*For 0–20x cycles*	73.11 ± 14.9	81.0 ± 10.6	69.9 ± 4.9

This change in diffusivity alters kinetics of immobilized LOx and the resulting oxygen consumption profiles. Increased swelling allows local lactate and O_2_ molecules to interact more readily with LOx active sites, encouraging more rapid enzyme saturation. As more O_2_ is consumed, BMAP is quenched less and therefore emits with a longer lifetime. Thus, the optical saturation is reached at lower bulk lactate concentrations. Higher pAam concentration increases enzyme-substrate contact, effectively lowering usable range of the device.

An interesting note is that *D_L_* values scale well with sensitivity metrics. Addition of only 10% pAam does little to increase lactate diffusion (and therefore sensitivity). pAam is much more hydrophilic than pHEMA, but is needed in higher ratios to significantly affect gel microstructure. Both sensitivity and *D_L_* metrics for the 75:25 pHEMA:pAam sensor are double what is seen in pure pHEMA gels, indicating promise for high measurement precision within a normal lactate range.

Response times calculated for sensor types were not statistically different between the materials. All materials were able to achieve a stable optical response within a 20-min window after introducing the step change in lactate level. This is similar to response times for current electrochemical-based sensors [56,57] and is considered adequate to effectively monitor changes in systemic conditions.

The 90:10 pHEMA:pAam and pure pHEMA sensors cover the normal physiological lactate range (0–30 mg/dL). Comparing sensors with those found in the literature indicates a general improvement in performance, even for these “disconnected” devices that are noninvasively interrogated. Garjonyte *et al.* and Palmisano *et al.* developed LOx-based amperometric biosensors with linear responses up to 7.2 and 1.8 mg/dL lactate, respectively [58,59]. Ibupoto *et al.* developed an electrochemical sensor with a high LOD_high_ (90 mg/dL) by immobilizing LOx on ZnO nanorods [60]. In regards to optically-based lactate sensors, Marquette *et al.* developed a fluorescent lactate sensor by co-immobilizing LOx and luminol onto the end of an optical fiber, although the LOD_high_ found was very low (30 pM) [61]. The system developed by Hu *et al.* had a calculated LOD_high_ of 45 nM [30]. While this list is not exhaustive, it shows that our platform competes and in several cases outperforms other lactate sensing modalities. Importantly, the platform we have shown here displays significantly higher LODs when compared to other optically-based lactate sensors found in the literature. It is these that would be potentially competing with our approach for fully-implanted sensing materials, if they could be modified to function in such a manner. While a select number of electrochemical lactate sensors have higher upper LODs, they still require a physical connection to transmit a signal whereas our system does not.

### 3.3. Acute Loss of Sensor Function

To test for acute reduction in sensor response to lactate, sensors were exposed to 20 consecutive lactate challenges. Lifetime values at 5, 10, and 20 lactate challenges were compared against initial flight plan lifetimes for freshly prepared sensors. Signal retention was calculated as a metric of the change in response after repeated exposures by determining the difference between the initial response lifetimes (Cycle 1). This metric is described in Equation (5).

(5) $$Signal\;retention\;\left(\%\right)=\left[\frac{\left({\text{τ}}_{cycle\;x20\;}–{\text{τ}}_{cycle\;01}\right)}{{\text{τ}}_{cycle\;x1}-{\text{τ}}_{cycle\;01}}\right]*100$$

where τ*_cycle x_*_20_ is the emission lifetime at cycle 20, τ*_cycle x_*_1_ is lifetime for same concentration on the initial cycle, and τ*_cycle_*_01_ is the baseline τ recorded at 0 mg/dL lactate for BMAP on the first cycle.

**Figure 4:**
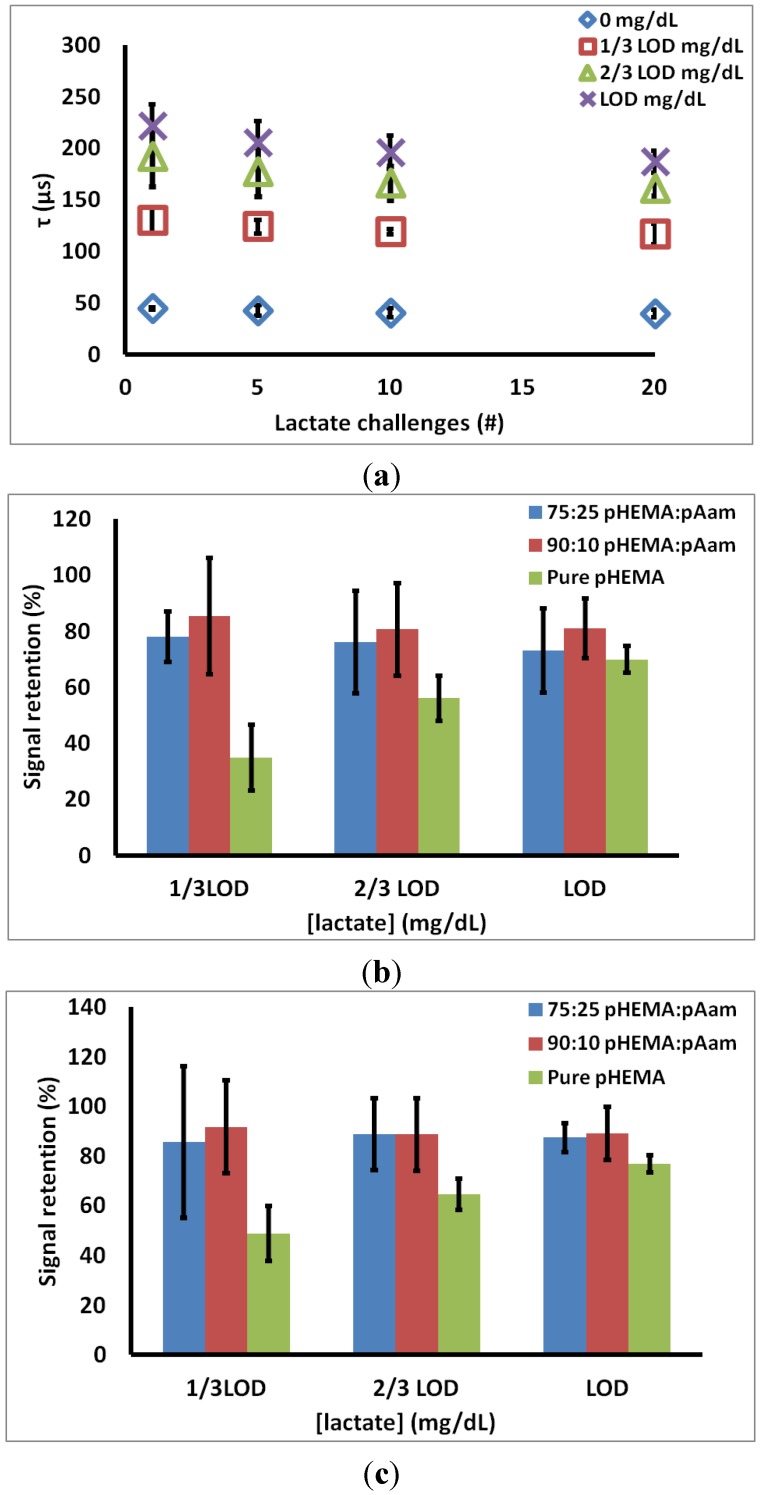
(**a**) 90:10 pHEMA:pAam signal retention over 20 cycles (**b**) % retention of first cycle signal (**c**) % retention of fifth cycle signal. Markers indicate average values, and error bars represent 95% confidence intervals between measured signal retention for *n* = 3 sensors.

Figure 4a contains representative data from the 90:10 pHEMA:pAam materials, indicating how the measured lifetimes at each lactate concentration changed over 20 cycles. Figure 4b is a summary of the signal retention over 20 cycles for all three sensor types. For 75:25 pHEMA:Aam sensors, signal retention of 78.1% ± 9.0%, 76.1% ± 18.4%, 73.1% ± 14.9% is seen at 1/3 LOD_high_, 2/3 LOD_high_, and LOD_high_, respectively. For 90:10 pHEMA:pAam sensors, signal retention of 85.5% ± 20.8%, 80.6% ± 16.5%, 81.0% ± 10.6% is seen at 1/3 LOD_high_, 2/3 LOD_high_, and LOD_high_, respectively. Finally, pure pHEMA sensors signal retention of 34.9% ± 11.8%. 56.1% ± 8.0%, 69.9% ± 4.9%, is seen at 1/3 LOD_high_, 2/3 LOD_high_, and LOD_high_, respectively.

Investigation of acute signal loss reveals an initial reduction in emission lifetime seen before a pseudo-stable response is reached, as there is no statistical change in lifetime after the 5th exposure cycle (Figure 4a). This “break-in” could be due to leaching of LOx not covalently bound to the hydrogel, though leaching studies done on freshly-prepared sensors showed no evidence of such (data not shown). The likely culprit is chemical and mechanical stresses induced on enzyme during polymerization and swelling during equilibration. These partially unstable proteins quickly denature (within first 2 h of lactate challenge), leaving remaining LOx to function properly. Figure 4c shows stability of lifetimes of each sensor type at LOD_high_ between cycles 5–20. After an initial loss in activity, all co-polymer materials maintained a stable response, with no statistical difference from the 5th cycle response to the 20th cycle at any of the test lactate concentrations.

These data also suggest a positive relationship between sensor stability and increasing pAam concentration. Both 75:25 pHEMA:pAam and 90:10 pHEMA:pAam experienced statistically similar signal retention throughout the study, while pure pHEMA formulations displayed higher levels of LOx activity loss (about 50%–70% retention between cycles 5–20). It follows that gels containing some acrylamide are better suited for retaining LOx activity, most likely due to the hydrophilic nature of pAam that yields an encapsulation more consistent with the enzymes’ native environment.

Aside from leaching and mechanical denaturation issues, neither of which were observed in our studies, enzyme activity is the primary issue for sensor stability. It is difficult to quantify enzyme activity within a semisolid medium. Furthermore, it is important to appreciate that, upon hydrogel immobilization, the kinetics of LOx no longer exclusively depend on lactate concentration. The LOx/polymer interface restricts transport, making substrate less available as when in solution. This means that the hydrogel composition will directly determine the rate of lactate delivery to the enzyme; this system requires normalizing conditions for direct comparison. Since each sensor type has a different calculated LOD_high_, we chose to expose them to the same normalized concentrations relative to this LOD during the experiment; this resulted in different absolute bulk lactate concentrations but effectively the same lactate flux. Although LOD_high_ was used in an attempt to normalize data, we recognize that dissimilar lactate challenges may provide for variable concentrations of substrate near immobilized LOx and therefore different reaction rates.

## 4. Conclusions

*In vitro* characterization of a novel biosensor designed for lactate detection has been described, using enzyme-oxygen, phosphor sensing chemistry immobilized within three different co-polymer formulations. The findings reveal that modification of co-polymer ratios allows for tunable macro sensor characteristics by controlling substrate diffusion through careful co-polymer selection. This matched expectations based on known hydrophilicity differences between the pHEMA and pAam materials studied, though the magnitude of the gains in oxygen diffusivity were surprisingly low. Furthermore, this tuning in response properties was possible by adjusting lactate diffusion with minimal effect on oxygen diffusion and phosphor sensitivity to oxygen.

While the hydrogel composition affected the transport properties, there was also an apparent change in acute stability that favors the incorporation of at least some acrylamide. Interestingly, sensor stability to repeated lactate challenges does not directly correlate with the diffusion properties studied herein. This suggests further investigation is necessary to understand mechanisms underlying sensor performance loss to allow optimal device design.

This study also highlights the potential of the enzyme-oxygen phosphor-hydrogel platform, as it is a modification of a previous glucose sensor. By switching the enzyme and adjusting the hydrogel properties, sensitive and stable lactate sensors were successfully developed. The same approach may, in principle, be used to extend the range of analytes to other targets using oxidoreductase enzymes.
